# Novel sequential therapy with metformin enhances the effects of cisplatin in testicular germ cell tumours via YAP1 signalling

**DOI:** 10.1186/s12935-022-02534-w

**Published:** 2022-03-09

**Authors:** Kancheng He, Zitaiyu Li, Kun Ye, Yihong Zhou, Minbo Yan, Hao Qi, Huating Hu, Yingbo Dai, Yuxin Tang

**Affiliations:** 1 Department of Urology, The Fifth Affiliated Hospital of Sun Yat-Sen University, Zhuhai, 519000 Guangdong China; 2 Guangdong Provincial Key Laboratory of Biomedical Imaging, The Fifth Affiliated Hospital, Sun Yat-Sen University, Zhuhai, 519000 Guangdong China; 3 State Key Laboratory of Quality Research in Chinese Medicine/Macau Institute for Applied Research in Medicine and Health, Macau University of Science and Technology, Avenida Wai Long, Taipa, Macao China

**Keywords:** Cisplatin, Metformin, Testicular germ cell tumour, Sequential treatment, YAP1

## Abstract

**Background:**

Testicular germ cell tumours (TGCTs) are the most commonly diagnosed malignancy in young men. Although cisplatin has been shown to be effective to treat TGCT patients, long-term follow-up has shown that TGCT survivors who accepted cisplatin treatment suffered from a greater number of adverse reactions than patients who underwent orchiectomy alone. As metformin has shown an anticancer effect in various cancers, we investigated whether metformin could enhance the effects of cisplatin to treat TGCTs.

**Methods:**

The anticancer effects of different treatment strategies consisting of metformin and cisplatin in TCam-2 and NTERA-2 cells were assessed in vitro and in vivo. First, we used a colony formation assay, CCK-8 and MTT assays to explore the viability of TGCT cells. Flow cytometry was used to assess the cell cycle and apoptosis of TGCTs. Then, Western blotting was used to detect the protein expression of TGCTs cells after different treatments. In addition, a xenograft model was used to investigate the effects of the different treatments on the proliferation of TGCT cells. Immunohistochemistry assays were performed to analyse the expression of related proteins in the tissues from the xenograft model.

**Results:**

Metformin inhibited the proliferation of TCam-2 and NTERA-2 cells by arresting them in G1 phase, while metformin did not induce apoptosis in TGCT cells. Compared with cisplatin monotherapy, the CCK-8, MTT assay and colony formation assay showed that sequential treatment with metformin and cisplatin produced enhanced anticancer effects. Further study showed that metformin blocked the cells in G1 phase by inducing phosphorylated YAP1 and reducing the expression of cyclin D1, CDK6, CDK4 and RB, which enhanced the chemosensitivity of cisplatin and activated the expression of cleaved caspase 3 in TGCTs.

**Conclusions:**

Our study discovers the important role of YAP1 in TGCTs and reports a new treatment strategy that employs the sequential administration of metformin and cisplatin, which can reduce the required cisplatin dose and enhance the sensitivity of TGCT cells to cisplatin. Therefore, this sequential treatment strategy may facilitate the development of basic and clinical research for anticancer therapies to treat TGCTs.

## Background

Testicular germ cell tumours (TGCTs) are the most commonly diagnosed malignancy in men aged 15 to 40 [1]. Although the incidence rate of TGCTs is only 2% in male malignancies, the morbidity has constantly increased over the last four decades [2]. TGCTs can be divided into seminomas, non-seminomas and mixed germ cell tumours by histologic subtype. At present, with the development of chemotherapy, the 5-year survival rate in men aged 15 to 39 is 87.5%, and that in men aged 40 to 69 is 72.3% [3]. However, a long-term cohort study showed that TGCT survivors who accepted cisplatin-based chemotherapy had a higher risk of vascular damage and cardiovascular morbidity in their long-term follow-up than survivors treated with orchiectomy alone [4]. In addition, the cumulative dose of cisplatin was closely related to peripheral neuropathy, ototoxicity and renal toxicity [5]. Moreover, cisplatin therapy for TGCTs has been linked with a dose-dependent increased risk of secondary malignancies, such as leukaemia and other solid cancers [6]. Hence, more effective strategies that enhance chemosensitivity and reduce the administered dose of cisplatin should be explored.

Metformin is a first-line oral drug for the treatment of type 2 diabetes mellitus. It can decrease blood glucose by enhancing insulin sensitivity and promoting glucose uptake into peripheral tissues. In recent years, various studies have reported that metformin may reduce the incidence of cancer and improve the prognosis of cancer patients [7–9]. For example, metformin was shown to inhibit the proliferation of breast cancer by decreasing the N6 methylation level [10]. Metformin also induces apoptosis of ovarian cancer cells via mitochondrial damage and endoplasmic reticulum stress [11]. Therefore, it seems that utilizing the anticancer effects of metformin may lead to a broadening of its applications to TGCT therapy.

The Hippo pathway plays an important role in cell proliferation and differentiation. Dysregulation of the Hippo pathway contributes to tumorigenesis. As the core factor of the Hippo pathway, Yes-associated protein (YAP1) acts as an oncogene that is activated in various human cancers [12–14]. YAP1 not only regulates the proliferation of cancer cells but also plays a central role in mediating resistance to cancer therapy, such as targeted therapies, chemotherapy, immunotherapies and radiotherapy [15–17]. Various studies have confirmed that metformin can sensitize tumour cells to antitumour drugs by inhibiting the expression level of YAP1 [18, 19].

Despite all of these anticancer functions that metformin has shown in various types of cancers, its anticancer ability against TGCTs has not yet been reported. In addition, there are no articles that have focused on the Hippo pathway and metformin in TGCTs. Therefore, in this study, we aimed to discover the correlation between metformin, the Hippo pathway and TGCTs.

## Methods

### Cell culture and treatment

The seminoma cell line TCam-2 was kindly donated by Professor Riko Kitazawa, Ehime University Hospital [20, 21]. The testicular embryonal carcinoma cell line NTERA-2 was obtained from the ATCC (Manassa, USA). All cells were free of mycoplasma contamination and used in experiments within 30 passages after thawing. TCam-2 cells were cultured in RPMI 1640 (Gibco, USA) with 10% foetal bovine Serum (FBS) (Gibco, USA) and 1% penicillin/streptomycin (Gibco, USA). NTERA-2 cells were cultured in complete DMEM (4.5 g/L glucose) (Gibco, USA) and incubated at 37 °C in a humidified atmosphere containing 5% CO_2_. In vitro, cells were treated with 5–20 mM metformin (KeyGEN, China). Additionally, TCam-2 cells were treated with 5 μM cisplatin, and NTERA-2 cells were treated with 0.1 μM cisplatin (SelleckChem). The treatment strategies included monotherapy (metformin or cisplatin monotherapy for 72 h), the combination therapy (synchronous treatment with metformin and cisplatin for 72 h) and sequential treatment. Specifically, sequential treatment with metformin and cisplatin was performed by treating TCam-2 and NTERA-2 cells with metformin for 48 h and then withdrawing it for 24 h before the cisplatin exposure for another 24 h. Verteporfin (VP), which was the YAP1 inhibitor, was purchased from MedChemExpress. Both TCam-2 and NTERA-2 were respectively treated with 3 μM VP for 48 h.

### Apoptosis analysis

Both TCam-2 and NTERA-2 cells (5 × 10^4^/well) were seeded in triplicate in RPMI 1640 and DMEM with FBS in 6-well plates and treated with metformin and cisplatin at the indicated concentrations separately or in combination. After treatment, the cells were washed, resuspended in binding buffer, and stained with Annexin V-FITC/ propidium iodide (PI) according to the manufacturer’s instructions (BD Biosciences). The stained cells were analysed by flow cytometry (CytoFLEX, Beckman Coulter, CA) and the percentage of cells in apoptosis stage was determined using FlowJo VX software. The histograms of apoptosis were represented and compared by GraphPad Prism 8 software.

### Cell cycle analysis

For cell cycle analysis, cells were harvested, washed with PBS, fixed with prechilled 70% ethanol, and maintained overnight at 4 °C. The fixed cells were then collected, washed and resuspended in PBS. The cells were incubated with 1 mg/mL RNase and 50 µg/mL PI for 30 min at 37 °C and subjected to flow cytometry analysis (Beckman Coulter, CA). The cell cycle results were analysed with ModFit LT 5.0 (Verity Software House). The histograms of each cell cycle phases were represented and compared by GraphPad Prism 8 software.

### Cell viability by MTT assay

Both TCam-2 and NTERA-2 cells were adhered in 96-well plates (3 × 103 cells) with standard culture medium for 24 h. Then cell medium was replaced with 100 μL fresh medium supplemented with different concentrations of metformin (1 mM, 5 mM, 10 mM, 15 mM, 20 mM). Standard medium served as a control. Following exposure to the solutions for different experimental time (24 h, 48 h and 72 h), 10 μL of MTT (Solarbio, China) were added in each well and were incubated for 4 h at 37℃ in dark. Then, we discarded the MTT and added 150 μL of dimethyl sulfoxide (DMSO) to detect the optical density (OD) at 490 nm with a microplate reader (EnVision 2105, PerkinElmer, England). In order to find the 50% inhibitory concentrations (IC50 values) of cisplatin monotherapy, combination therapy (metformin + Cisplatin) and sequential treatment (metformin − cisplatin), the Pearson Correlation coefficient was calculated for showing the correlation between viability of cells and therapy strategies. All the data were calculated by GraphPad Prism 8.

### Cell proliferation by CCK-8 assay

A cell counting kit-8 (Fude Biological Technology, China) was used for this assay, after 24 h, 48 h, 72 h, 96 h and 120 h of growing TCam-2 and NTERA-2 cells in experimental medium. To evaluate the proliferation of TCam-2 and NTERA-2 cells, 150 μL of cell suspension (3 × 10^3^ cells) was cultivated in three 96-well plates with 10 mM metformin. A total of volume of 15 μL CCK-8 solution was added to each well in different experimental time (24 h, 48 h, 72 h, 96 h and 120 h). The plates were then incubated at 37 °C for an additional 90 min. The OD at 450 nm was measured with a microplate reader.

### RNA-seq data acquisition and bioinformatics analysis

The transcriptome profile of TGCTs was downloaded from the TCGA database in March 2021. The RNA-seq data of normal testicular tissues were downloaded from the Genotype-Tissue Expression (GTEx) project. Then, we converted the downloaded data from level 3 HTSeq fragments per kilobase per million to transcripts per million formats for further analysis. The differential expression of YAP1 was analysed by the wilcoxon rank sum test and visualized with the beeswarm package of R software.

### Quantitative real-time PCR (qRT–PCR)

Total RNA was extracted from TCam-2 and NTERA-2 cells by using Total RNA Kit (Omega Biotek, China). The mRNA expression was measured in triplicate using SYBR Green qPCR Mix (Vazyme, China) according to the manufacturer’s instructions. Primer sequences were as follows: YAP1: Forward: 5’TAGCCCTGCGTAGCCAGTTA, Reverse: 5’TCATGCTTAGTCCACTGTCTGT. βactin: Forward: 5’TGACGTGGACATCCGCAAAG. Reverse: 5’CTGGAAGGTGGACAGCGAGG. The comparative Ct method was used to calculate the relative mRNA expression, and β-action was used as an internal control.

### Western blot analysis

For the immunoblotting assay, the protein β-actin (AC026, ABclonal) was used as a loading control. The primary antibodies were as follows: anti-YAP1 (A1001, ABclonal), anti-active-Caspase 3 (A11021, Abclonal), anti-pro Caspase 3 (A2156, Abclonal), anti-CDK6 (14052-1-AP, Proteintech), anti-phospho-YAP1-S127 (AP0489, ABclonal), anti-CDK4 (A11136, ABclonal), anti-Cyclin D1 (A19038, ABclonal) and anti-RB (A17005, ABclonal). The protein bands were visualized by ECL (Affinity, China) and analysed by iBright 1500 (Thermo, USA).

### Immunofluorescence assay

Both TCam-2 and NTERA-2 cells were fixed with 4% paraformaldehyde (Macklin, China) for 5 min and permeabilized with 0.1% Triton X-100 (Sigma–Aldrich) for 5 min at room temperature. After that, the cells were blocked with 5% BSA in PBS for 1 h and incubated with primary antibodies against YAP1 (1:100, ABclonal). Cells were then labelled with FITC goat anti-rabbit IgG (1:200, Earthox). Nuclei were stained with DAPI (1:400, Solarbio). Fluorescence images were acquired by using an inverted fluorescence microscope (Olympus).

### Colony formation assay

TCam-2 cells and NTERA-2 cells (500/well) were seeded in 6-well plates and treated with metformin and cisplatin. The medium was replaced every 3 days until the TCam-2 and NTERA-2 cells in the control wells reached 80–100% confluence. Then, the cells were fixed with 4% paraformaldehyde (Macklin, China) for 20 min and stained with 1% crystal violet for 10 min.

### Xenografts

This study was approved by the Ethics Committee of the Fifth Hospital of Sun Yat-sen University, Zhuhai, Guangdong Province, China. The in vivo experiments were also conducted in accordance with the Guide for Care and Use of Laboratory Animals published by the United National Institutes of Health. Male BALB/c nude mice were purchased from Guangdong Medical Laboratory Animal Center. Animals were maintained under pathogen-free conditions in the Guangdong Provincial Key Laboratory of Biomedical Imaging. Sixteen male BALB/c nude mice were subcutaneously injected with TCam-2 seminoma cells and NTERA-2 embryonal carcinoma cells near the limbs to establish xenografts (1 × 10^6^/mouse, 0.2 ml) at each injection site. After one week, mice bearing engrafted tumours with a volume of 50 mm^3^ were randomized to receive either oral treatment with 200 mg/kg metformin (n = 4), intraperitoneal injection of 6 mg/kg cisplatin (n = 4), sequential treatment of metformin and cisplatin (n = 4) or PBS treatment (n = 4) according to the dosing schedule provided in Fig. 5A. The perpendicular tumour diameters were measured with callipers. Tumour volumes were calculated as (length × width^2^)/2 every three days. Tumours were weighed after the mice were euthanized by cervical dislocation. All nude mice were sacrificed when the tumour measurement exceeded 20 mm in any one dimension. Tumours were fixed in 4% paraformaldehyde and embedded in paraffin.

### Immunohistochemistry (IHC)

Tumour sections from the xenografts were deparaffinized in xylene, rehydrated in ethanol and finally rehydrated in double‐distilled water. The sections were then placed in 0.01 mol/L citrate buffer (pH 6.0) and heated in a microwave for 20 min to retrieve antigens. The sections were blocked with 3% goat serum for 60 min and then incubated with anti-phospho-YAP1-S127 (1:100, ABclonal) and anti-RB (1:100, ABclonal) overnight at 4 °C and stained with 3,3’-diaminobenzidine. Densitometry analysis was performed by ImageJ.

### Statistical analysis

All experiments were repeated in triplicate, and all data are presented as the average of three independent experiments. Data were analysed with GraphPad Prism version 8.0 and are presented as the mean ± standard deviation (SD). Student’s t test was used to analyse the statistical significance of the differences between two groups. One-way ANOVA was performed for statistical significance among multiple groups.

## Results

### Metformin inhibited the proliferation of TCam-2 and NTERA-2 cells by inducing G1 phase arrest without apoptosis

**Fig. 1:**
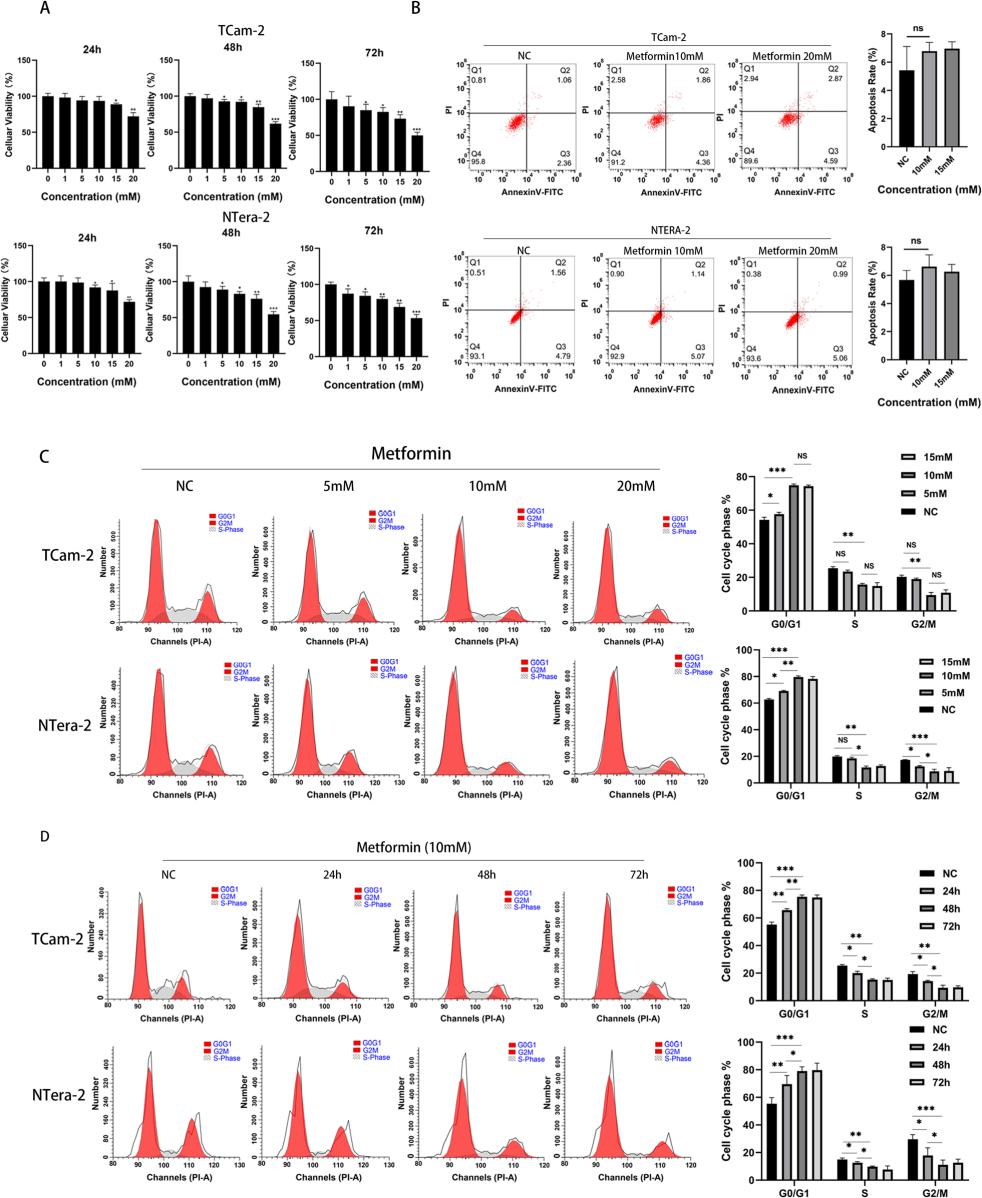
**A** Cell viabilities of TCam-2 and NTERA-2 cells determined by MTT assay after treatment with different concentrations of metformin (1 mM, 5 mM, 10 mM, 15 mM and 20 mM) for 24 h, 48 h and 72 h. **B** Flow cytometry analysis showed the cellular apoptosis of TCam-2 and NTERA-2 cells treated with 10 and 20 mM metformin at 72 h, and the average percentages of apoptotic cells were determined. **C** Flow cytometry showed that the TCam-2 and NTERA-2 cell cycle was blocked in G1 phase after treatment with metformin at 5–20 mM for 72 h. **D** TCam-2 and NTERA-2 cells were treated with metformin for 24, 48 and 72 h. Student’s t-test (two-tailed, equal variance) was used to calculate P values: *P < 0.05, **P < 0.01, ***P < 0.001 and ****P < 0.0001

The human-derived seminoma cell line TCam-2 and the testicular embryonal carcinoma cell line NTERA-2 were used to assess the effects of metformin on the proliferation of TGCTs. Both TCam-2 and NTERA-2 cells were treated with metformin at different concentrations (5–20 mM). According to our results, the antiproliferative effects of metformin occurred in dose- and time-dependent manners in vitro. During the first 24 h, 15 mM metformin inhibited the proliferation of TCam-2 cells, while the smallest concentration required to inhibit in NTERA-2 cells was 10 mM. At 72 h, the viability of TCam-2 and NTERA-2 cells was significantly inhibited when the concentration of metformin exceeded 5 mM (Fig. 1A).

To further investigate the inhibition mechanisms of metformin, flow cytometry was used to assess whether metformin induced TCam-2 and NTERA-2 cell apoptosis. As shown in Fig. 1B, a high concentration of metformin did not induce cellular apoptosis in TCam-2 or NTERA-2 cells after continuous application for 72 h. Therefore, we next investigated the cell cycle in these metformin-treated cells. Compared with the control groups, metformin significantly induced G1 phase arrest in TCam-2 and NTERA-2 cells at concentrations ranging from 5 to 20 mM. In addition, as the concentration of metformin increased, its effects on cell cycle arrest improved, while the cell cycle arrest ability of 20 mM metformin showed the same trends as that of 10 mM metformin (Fig. 1C). As shown in Fig. 1D, with prolonged metformin monotherapy, its effect on TCam-2 and NTERA-2 cell cycle arrest in G1 phase gradually increased, while the proportions of cells in G2/M and S phase gradually decreased. The dynamic change in the cell cycle showed that after incubation for 48 h, the proportion of TCam-2 and NTERA-2 cells in G1 phase reached their peak, displaying the same trends as the G1 proportions at 72 h.

### Sequential treatment with metformin and cisplatin inhibited TCam-2 and NTERA-2 cell function in vitro

**Fig. 2:**
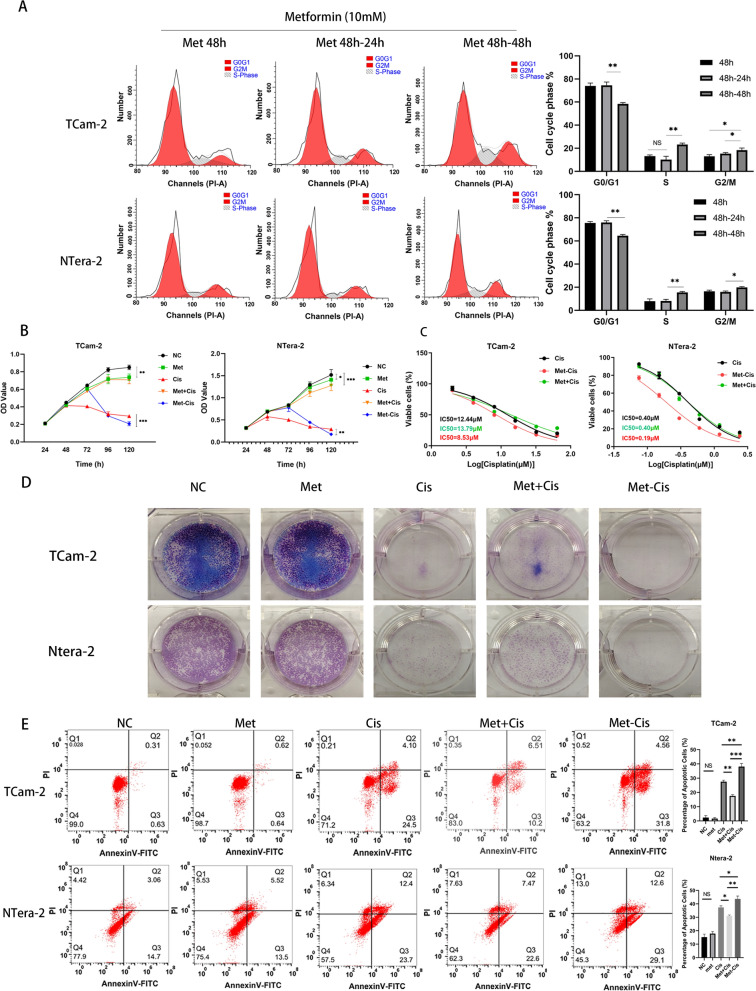
**A** TCam-2 and NTERA-2 cells were treated with metformin for 48 h, and flow cytometry was used to assess the changes in the cell cycle during withdrawal at 24 and 48 h. **B** The viability of TCam-2 and NTERA-2 cells after exposure to the five treatment strategies was determined by CCK-8 assay. **C** The IC50 of cisplatin was determined in TCam-2 and NTERA-2 cells treated with combination treatment (metformin + cisplatin) and sequential treatment (metformin  − cisplatin). **D** A colony formation assay was used to detect the anticancer effects of the different treatment strategies. **E** Flow cytometry was used to detect the effects of apoptosis of the different treatment strategies. The average percentage of TCam-2 and NTERA-2 cell apoptosis was quantified

Owing to the cell cycle arrest ability of metformin, we next investigated the effects after withdrawal of metformin in TCam-2 and NTERA-2 cells and found that as the withdrawal time was prolonged to 48 h, the proportion of cells in G1 phase gradually decreased, while the proportion of cells in S and G2/M phase increased. This result showed that metformin could arrest the cell cycle in G1 phase and withdrawing metformin led to cell cycle recovery (Fig. 2A).

Based on above results, Sequential treatment with metformin and cisplatin was performed by treating TGCT cells with metformin for 48 h and then withdrawing it for 24 h before cisplatin exposure. According to Fig. 2B, sequential treatment with metformin followed by cisplatin showed a brilliant anticancer effect on the viability of both TCam-2 and NTERA-2 cells, while combination treatment with metformin and cisplatin may impede the anticancer effects of cisplatin. To further investigate whether these two treatment strategies affect the chemosensitivity of TCam-2 and NTERA-2 cells to cisplatin, we calculated the IC50 values of cisplatin from these two strategies. The IC50 values of cisplatin in TCam-2 and NTERA-2 cells were 12.44 μM and 0.4 μM, respectively, which were similar to the IC50 values of cisplatin in previous reports [22, 23]. Compared with the monotherapy group and combination group, sequential treatment reduced the IC50 of cisplatin in both TCam-2 and NTERA-2 cells. However, the simultaneous combination with metformin and cisplatin increased the IC50 values of cisplatin and reduced the chemosensitivity of TCam-2 cells. Besides, the simultaneous combination of metformin and cisplatin did not affect the IC50 values of cisplatin of NTERA-2 cells (Fig. 2C). The results from the colony formation assay confirmed our above finding, as sequential treatment efficiently inhibited colony formation of both TCam-2 and NTERA-2 cells. (Fig. 2D).

The effects of sequential treatment on apoptosis are shown in Fig. 2E. Apoptosis in TCam-2 and NTERA-2 cells treated sequentially was significantly elevated compared with cells treated with cisplatin alone and combination treatment. Moreover, the combination treatment with metformin and cisplatin had a lower apoptosis rate than the monotherapy cisplatin group in both TCam-2 and NTERA-2 cells.

### YAP1 was highly expressed in TGCTs, and metformin regulated the cell cycle in TCam-2 and NTERA-2 cells by phosphorylating the YAP1 protein

**Fig. 3:**
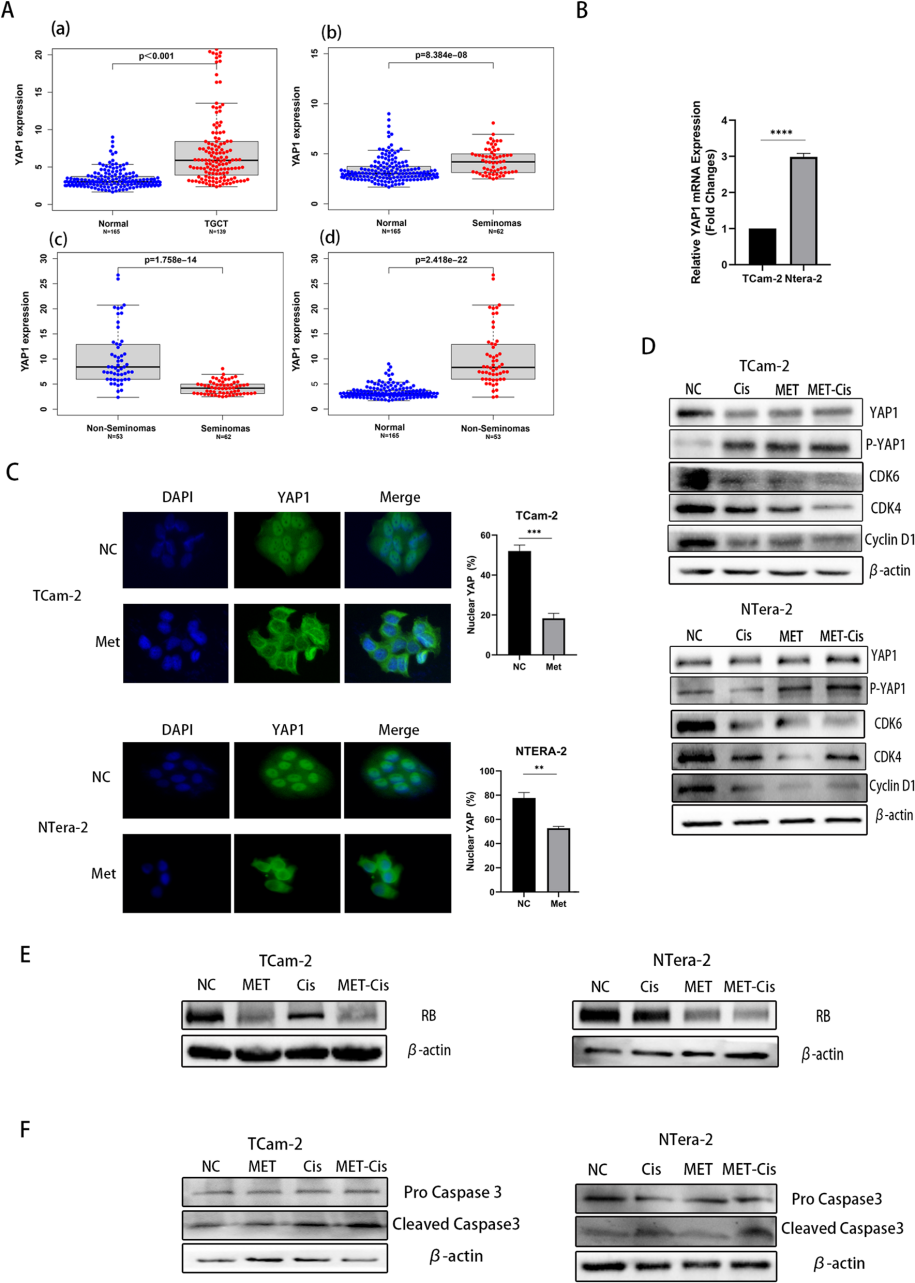
**A** The differential expression of YAP1 in 139 tumours and 165 normal testicular tissues visualized by using RNA-seq data from TCGA and GTEx (**a**). The differential expression of YAP1 in 165 normal tissues and 62 seminomas (**b**). The differential expression of YAP1 in 53 non-seminomas and 62 seminomas (**c**). The differential expression of YAP1 in 165 normal tissues and 53 non-seminomas (**d**). **B** The mRNA expression of YAP1 in TCam-2 and NTERA-2 cells. **C** Immunofluorescence analysis was used to assess the expression of YAP1 in TCam-2 and NTERA-2 cells treated with metformin. Images were captured at 200 × with an Olympus microscope. The percentage of cells with YAP localized in the nucleus (n ≥ 30 cells). **D**–**F**. Sequential treatment with metformin and cisplatin enhanced the effects of cisplatin through the YAP1/CDK6/CDK4/cyclinD1/RB axis and Caspase 3. Western blotting was used to assess the expression of related proteins

Metformin was reported to inhibit the proliferation of some cancer cells via the YAP1 pathway, which is the key factor in the Hippo pathway. However, the expression level of YAP1 in seminomas and non-seminomas is still unclear. To determine the difference in YAP1 expression between normal testicular tissue and TGCTs, we analysed its expression levels in 139 TGCT tissues and 165 adjacent testicular tissues and found that YAP1 was upregulated in TGCT tissues (P < 0.001, Fig. 3Aa). Moreover, we separated 62 seminomas, 53 non-seminomas and 24 mixed tumours from these 139 TGCT tissues. According these results, both seminoma and non-seminoma tissues had higher expression levels of YAP1 than the adjacent testicular tissues (Fig. 3Ab, Ad). Compared with the non-seminomas, a lower expression level of YAP1 was detected in seminomas than the non-seminomas ones (Fig. 3Ac). Our qPCR results also confirmed that the YAP1 levels were higher in the non-seminoma NTERA-2 cells than in the seminoma TCam-2 cells (Fig. 3B).

**Fig. 4:**
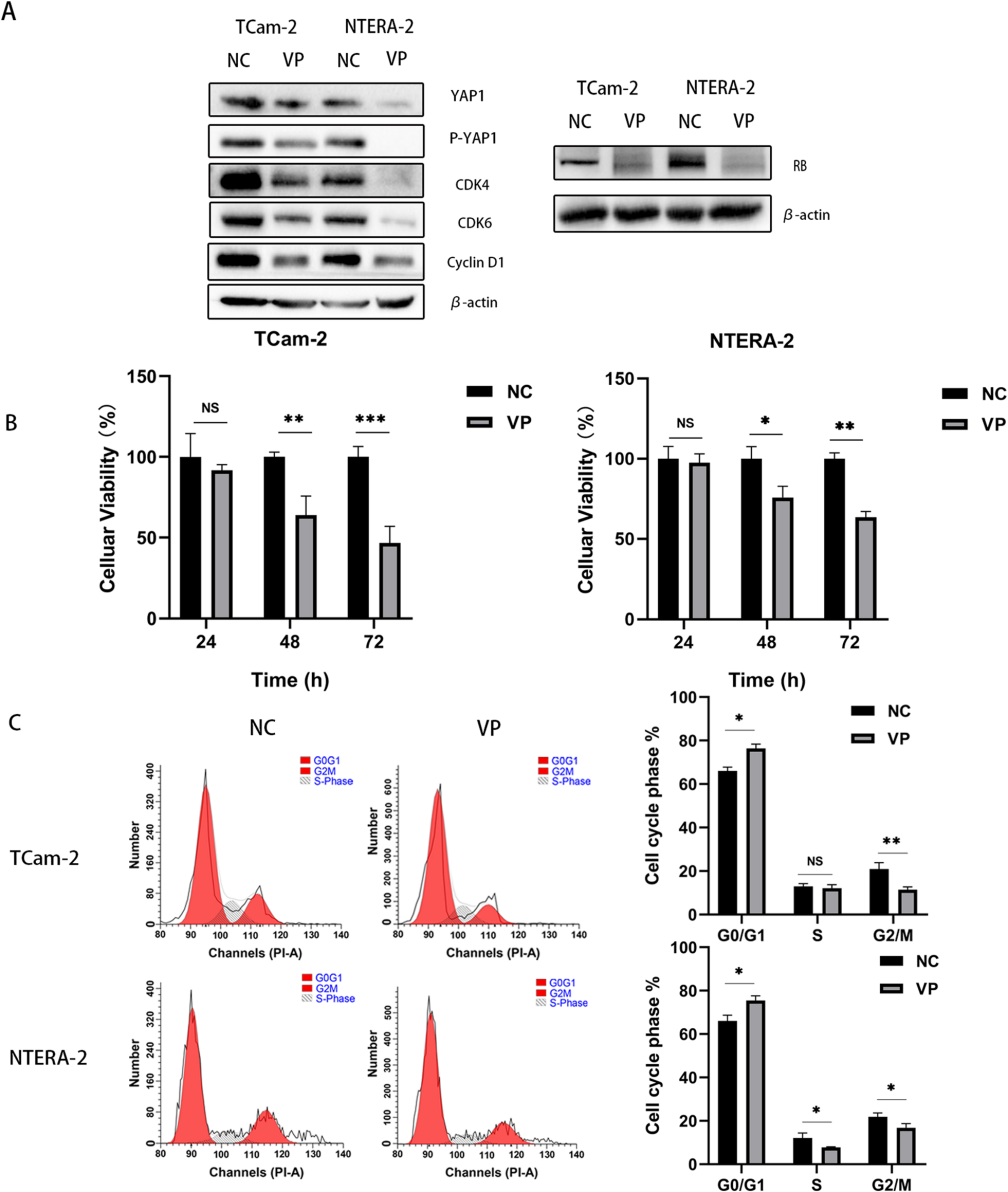
**A** The expression levels of YAP1, P-YAP1, CDK6, CDK4, CyclinD1 and RB were determined by Western blot analysis after treatment with VP. **B** The viability of TCam-2 and NTERA-2 cells treated with 3 μM VP was determined using the MTT assay. **C** The cell cycle was assessed in TCam-2 and NTERA-2 cells treated with 3 μM VP for 48 h

Consistent with the phosphorylation of YAP1, immunofluorescence staining revealed that metformin enhanced the cytoplasmic accumulation of YAP1 in TCam-2 and NTERA-2 cells. YAP1 showed a lower located in the nucleus decreased from 52 to 18% for NC versus Met in TCam-2 and from 78 to 53% for NC groups versus Met groups in NTERA-2, respectively (Fig. 3C). Western blot analysis showed that sequential treatment with metformin and cisplatin significantly induced YAP1 phosphorylation at S127 and led to the inhibition of G1 phase-related proteins, including CDK6, CDK4, cyclinD1 and RB. The expression of RB was also decreased by metformin and sequential treatment in TCam-2 and NTERA-2. (Fig. 3D, E). In addition, our results revealed that this novel sequential treatment strategy enhanced chemosensitivity to cisplatin and increased apoptosis in TCam-2 and NTERA-2 cells by activating the expression of cleaved caspase 3 (Fig. 3F). Moreover, compared to the monotherapy cisplatin group, the expression of cleaved caspase3 was significantly increased in the sequential group of both TCam-2 and NTera-2, which was similar to our flow cytometry apoptosis results. We further examined whether downregulation of YAP1 could lead to G1 phase arrest. We used VP, a pharmacological inhibitor of YAP, to treat TGCT cells. Western blot analysis found that VP not only downregulated YAP1 but also decreased the expression of p-YAP1, cyclinD1, CDK4, CDK6 and RB. (Fig. 4A). After cultured with VP for 48 h, cell viability and cell cycle analysis showed that VP inhibited the proliferation of both TCam-2 and NTERA-2 cells by inducing G1 phase arrest, which was similar to the cell cycle blockade caused by metformin (Fig. 4B, C).

### Sequential metformin-cisplatin treatment effectively inhibited the proliferation of TCam-2 xenografts in vivo

**Fig. 5:**
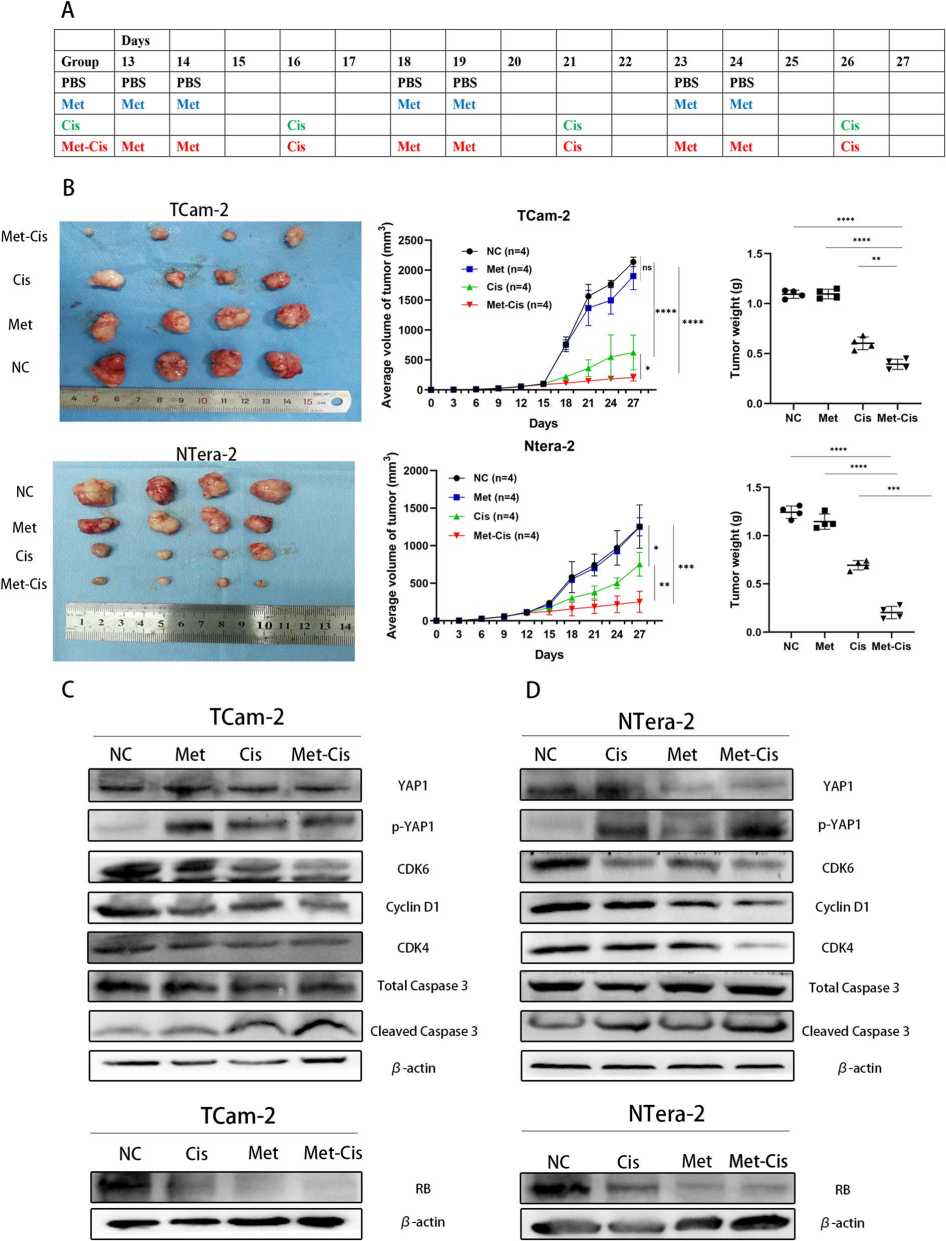
**A** Schedules of the four different treatment strategies. **B** Randomly grouped nude mice bearing TCam-2 and NTERA-2 tumours were treated with PBS (NC), Met (200 mg/kg), Cis (5 mg/kg) or metformin-cisplatin sequential treatment (Met-Cis) for three cycles. Representative tumours in nude mice from different groups. Tumour volumes were measured every 3 days, and the tumour weights were determined immediately after harvesting the whole tumours. **C**, **D** The expression levels of YAP1, P-YAP1, CDK6, CDK4, cyclinD1, RB, caspase 3 and cleaved caspase 3 were determined by Western blot in tumour tissues from the xenograft mice treated with different treatments

**Fig. 6:**
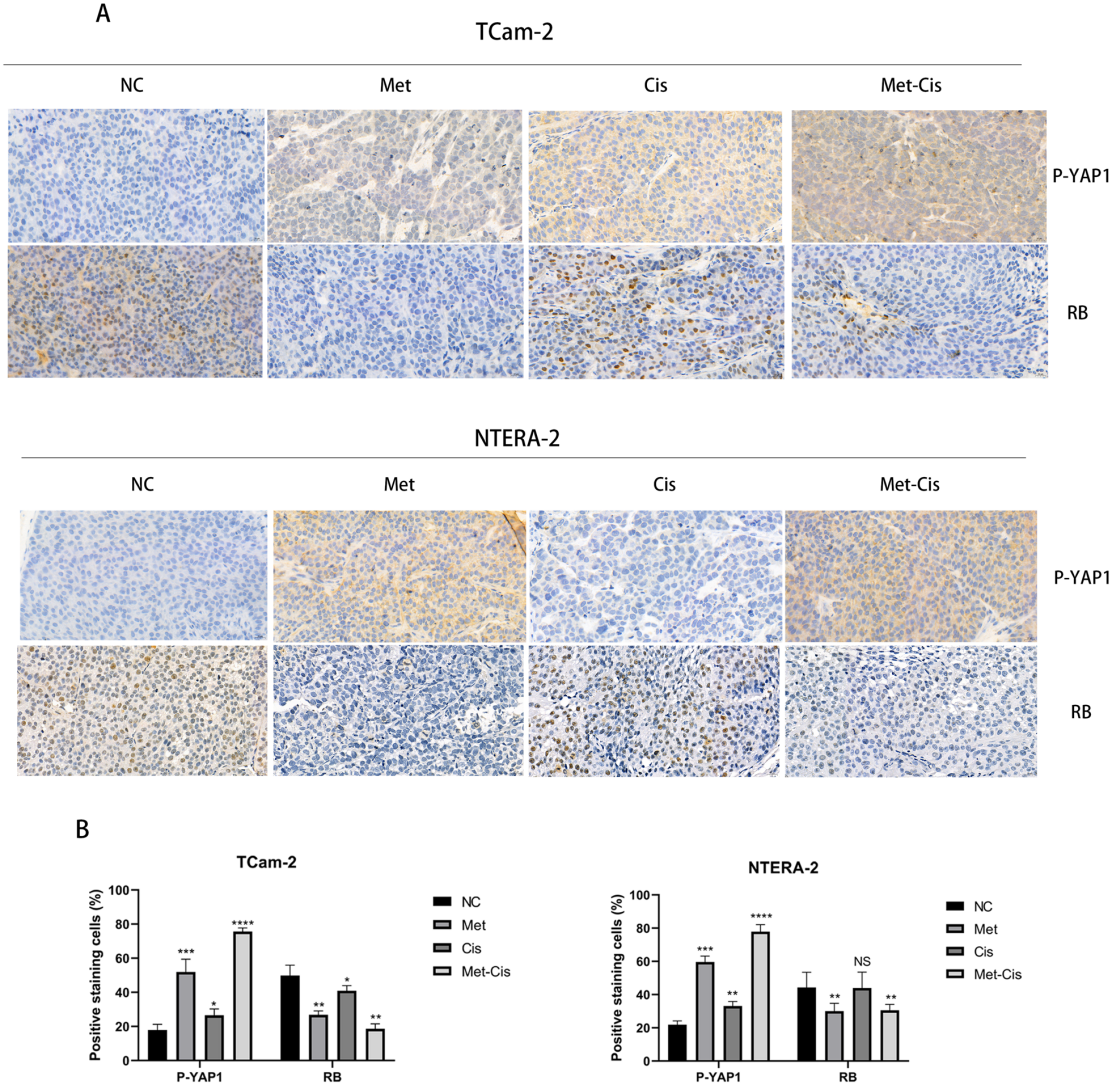
**A** The expression levels of P-YAP1 and RB were investigated by immunohistochemical staining of the tumour tissue from xenografts. Images were captured at 400 × with an Olympus microscope. **B** The percentages of p-YAP1 and RB positive cells and their staining intensities were assessed by ImageJ

**Fig. 7:**
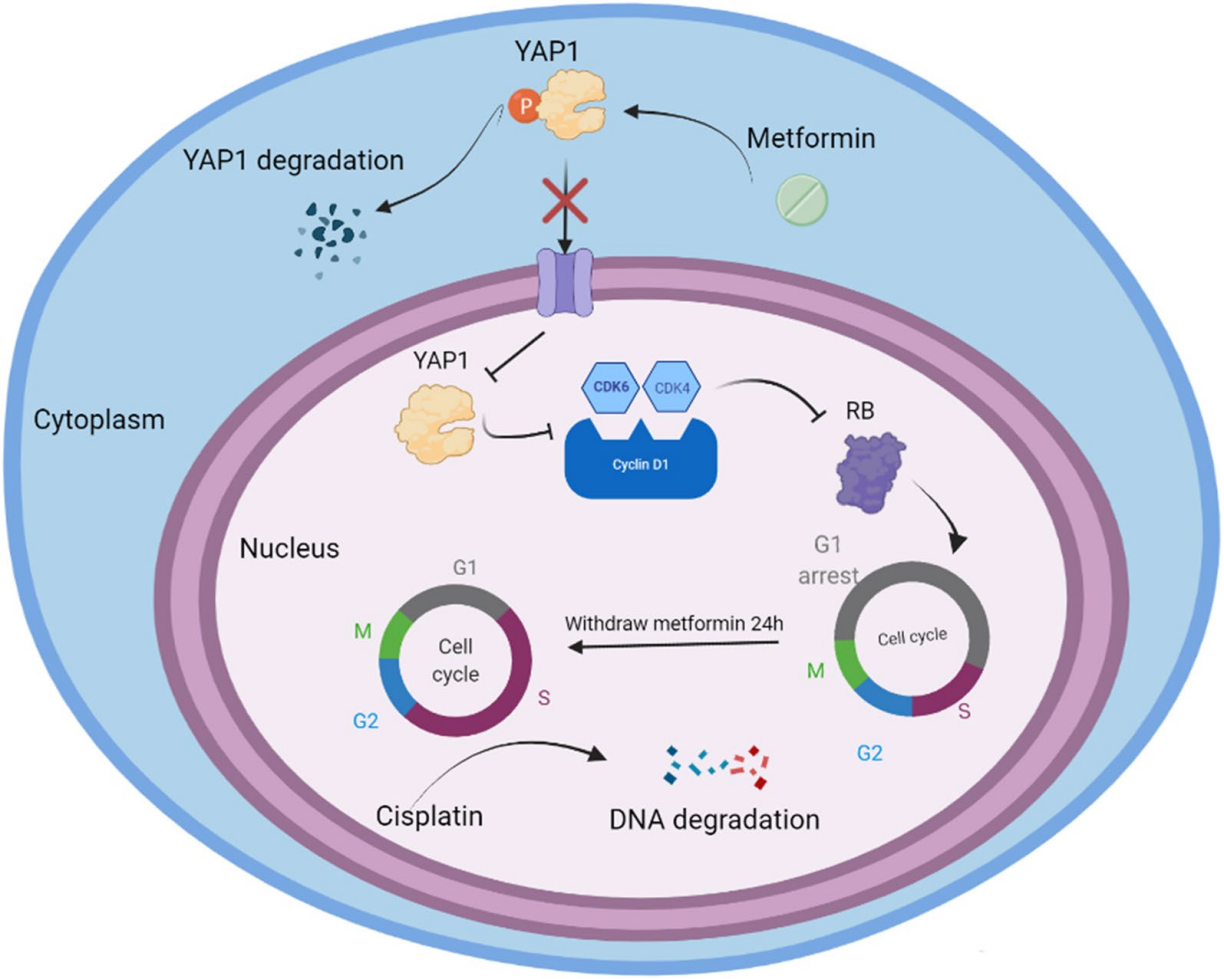
Proposed model for the mechanism of sequential metformin and cisplatin treatment. Metformin directly phosphorylated YAP1 and inhibited the translocation of YAP1 from the cytoplasm to the nucleus. Low nucleus protein of YAP1 decreased expression of cyclinD1, CDK6, CDK4 and led to G1 phase arrest in TCam-2 and NTERA-2. After withdrawing the metformin for 24 h, most of the cells were synchronized and ready to enter S and G2 phase, which enhanced their chemosensitivity to cisplatin. Therefore, sequential treatment (metformin-cisplatin) could enhance the chemosensitivity of cells and induce apoptosis

To further verify the efficiency of sequential treatment in vivo, we also established TGCT xenograft in nude mice models. After modelling for 7 days, all the nude mice were divided into four groups, and their treatment schedules are shown in Fig. 5A. The tumour volumes and weights in the sequential treatment group were significantly lower than those in the other groups (Fig. 5B). Western blot analysis showed a clear increase in the expression of phosphorylated YAP1 proteins, and the expression levels of CDK6, CDK4, cyclin D1 and RB were significantly reduced compared with those in the control groups (Fig. 5C, D). Similar to the in vitro results, the cleaved caspase 3 expression was highest in the sequential group, due to the cell cycle arrest of metformin and the sequential treatment with cisplatin. In addition, our IHC showed that metformin reduced the expression of YAP1 in nucleus, and increased the expression of p-YAP1 in both TCam-2 and NTERA-2 (Fig. 6A, B). Furthermore, the expression of RB in the metformin and sequential treatment group were significantly lower than that in the monotherapy cisplatin group and NC group (Fig. 6A). All of these in vivo and in vitro experimental results indicated that metformin inhibited the cell cycle via the YAP1/cyclinD1/CDK6/CDK4/RB pathway and increased cisplatin-induced apoptosis of both TCam-2 and NTERA-2 cells. The proposed mechanistic model is shown in Fig. 7.

## Discussion

Combination and sequential therapy that relies on complementary mechanisms of antitumour activity has gradually become a hotspot in cancer treatment. Currently, an increasing number of targeted therapies, including ALK, CDK and EGFR inhibitors combined with conventional radiotherapy and chemotherapy regimens are acknowledged as significant cancer treatment methods for individual therapy and to combat drug resistance [24, 25]. Moreover, combination treatments could maintain chemosensitivity and reduce the dose of chemotherapeutics and adverse reactions [26, 27]. However, Patrick et al. held different idea that compared with carboplatin monotherapy, the therapeutic effects of carboplatin and palbociclib combination treatment decreased substantially [28]. The above results indicate that combination treatment may not necessarily enhance the anti-cancer effect of chemotherapeutics in all cancers. Different tumour types, different chemosensitizers and different timing strategies for combination treatment regimens would lead to different effects in cancer treatment.

Various studies have extensively reported the effects of metformin for cancer prevention and therapy [29, 30]. Functioning as an antitumour drug, metformin has been reported to block precancerous lesions from progressing to become invasive in rectal cancer and bladder cancer [18, 31]. Further research found that the anticancer effects of metformin could not only suppress the proliferation of cancer cells but also induce apoptosis and inhibit the progression of tumour stem cells [32, 33]. In our study, we investigated the anti-cancer effects of various metformin and cisplatin treatments on TGCTs. We first compared some common treatment strategies and found that a sequential regimen of metformin and cisplatin showed a better treatment benefit than the other regimens, such as metformin monotherapy, cisplatin monotherapy and combination therapy (metformin + Cisplatin). Besides, the combination treatment with metformin and cisplatin had a lower apoptosis rate than the monotherapy cisplatin group in both TCam-2 and NTERA-2 cells. During our study, we confirmed that metformin can result in cell cycles retarding at G1 phase. Cisplatin causes much of its damage in S phase as cells try to undergo DNA replication with platinum cross-linked to their DNA, which was considered as the primary mechanism for tumour-specific killing. However, during the G1 phase arrest, both base excision and nucleotide excision repair could affect the effect of platinum–DNA adducts and decreased cisplatin-sensitivity [34, 35], which explain why the combination therapy had a lower apoptosis rate than the monotherapy cisplatin group. Therefore, we considered that when metformin was used for 48 h and then withdrawn for 24 h, most of the cells were synchronized and ready to enter S and G2 phase, which enhance their sensitivity to cisplatin. Our in vivo studies also confirmed that metformin and cisplatin sequential treatment markedly activated the expression of cleaved Caspase 3 and inhibited the growth of seminoma and non-seminoma cancers in xenograft models. Moreover, the treatment dose in our in vitro study was 200 mg/kg per day, which was consistent with the clinical dose of 500–2000 mg/d in humans [36]. This result verified that although metformin monotherapy could not induce the apoptosis of seminoma and non-seminoma cells, a sequential regimen with metformin could improve the effect of cisplatin, leading to TGCT cell apoptosis.

As an excellent adjuvant for chemotherapy, metformin could amplify the effects of cisplatin, which could decrease the cisplatin dose required and reduce its side effects [37]. The cumulative dose of cisplatin is closely related to various adverse reactions, such as cognitive decline, sexual dysfunction and chronic renal failure [38–40]. To minimize these cumulative-dose-dependent chemotherapy- induced toxicities, reducing chemotherapy therapeutic dose via combination and sequential treatment with low toxicity agents has been discovered. Adebayo et al. found that sequential treatment with ovatodiolide and doxorubicin increased anticancer effect of doxorubicin (IC50 = 4.4 μM), compared to simultaneous treatment with doxorubicin (IC50 = 10.6 μM) and doxorubicin alone (IC50 = 9.4 μM). Moreover, intracellular accumulations of doxorubicin were the least in sequential treatment with ovatodiolide and doxorubicin, when compared with doxorubicin or simultaneously treated cells [41]. With the aid of sequential strategy with metformin, the IC50 of cisplatin decrease in both TCam-2 and NTERA-2. Therefore, TGCT patients, who accept the sequential treatment, may receive the same therapeutic benefit from a lower dose of cisplatin and decrease their risk of related adverse reactions.

As a key oncogenic mediator in Hippo signalling, YAP1 is highly expressed in various cancers and maintains cancer growth and invasion activities [42, 43]. Therefore, many findings have suggested that suppressing YAP1 can impede tumour proliferation and migration [44, 45]. In our study, we first compared YAP1 expression between seminoma and non-seminoma and found that the expression of YAP1 in NTERA-2 cells was significantly higher than that in seminoma cells, which is consistent with the TCGA database results. Metformin has been reported to be a YAP1 inhibitor in various cancers that can directly phosphorylate YAP1 and inhibit the translocation of YAP1 from the cytoplasm to the nucleus [46, 47]. Moreover, some studies also found that YAP1 was closely correlated to the proteins of the cell cycle [48–50]. Thus, we assessed the expression level of YAP1 in seminoma and non-seminoma cells and found that it played an important role in the cell cycle of TGCTs. As the phosphorylation level of YAP1 increased, the expression of cell cycle-related proteins, such as cyclinD1, CDK6, CDK4 and RB, decreased. All of these proteins are key factors that directly regulate the G1 phase during the development of various cancers, including bladder cancer, prostate cancer and breast cancer [51–53]. Furthermore, the expression of RB is significantly decreased with the metformin treatment in TGCTs. As we all known that, RB is one of the most important tumour suppressors in various cancers [54]. However, numbers of clinical and basic studies also claimed that loss of RB function led to the efficient and sensitive response to chemotherapy in many cancers [55]. RB-deficient cells are more sensitive to DNA-damaging agents [56–58]. Similarly, a multicentre clinical study also confirmed that patients with RB deficiency predicts sensitive and efficient response to platinum-based chemotherapy in pancreatic neoplasia [59]. During this study, we found that metformin could reduce the expression of RB, which also increase the cisplatin chemosensitivity of TCam-2 and NTERA-2. Taken together, our study reports that treatment with metformin could induce G1 phase arrest in TCam-2 and NTERA-2 cells by promoting the phosphorylation of YAP1 and reducing the expression of cyclinD1/CDK6/CDK4/RB signalling. Therefore, YAP1 may be a potential therapeutic target for inhibiting TGCT cell proliferation.

The Hippo signalling pathway is closely related to cancer characteristics and clinical prognoses, including cancer cell proliferation, metastasis and apoptosis. This is the first study to investigate the correlation between metformin, Hippo signalling and TGCTs. Our data revealed that YAP1/CDK6/CDK4/cyclinD1/RB could be a potential therapeutic axis in TGCTs and sequential regimens of metformin and cisplatin could be a useful therapy for human seminomas and non-seminomas.

## Conclusions

Overall, our study showed that sequential regimens of metformin and cisplatin could enhance the sensitivity of both TCam-2 and NTERA-2 cells to cisplatin, which is closely related to the YAP1/CDK6/CDK4/cyclinD1/RB therapeutic axis. These findings are useful not only for comprehending the mechanism between cell cycle regulation and tumorigenicity but also for discovering that the YAP1 and Hippo pathways play an important role in the proliferation of TGCTs, which may facilitate the development of basic research and anticancer therapies against TGCTs.

## Data Availability

All data generated or analyzed during this study are included in this article.

## Abbreviations

TGCT: Testicular germ cell tumor
YAP1: Yes-associated protein
SD: Standard deviation
GTEx: Genotype-tissue expression project
TCGA: The cancer genome atlas
